# Development of Finite Element Models of PP, PETG, PVC and SAN Polymers for Thermal Imprint Prediction of High-Aspect-Ratio Microfluidics

**DOI:** 10.3390/mi13101655

**Published:** 2022-09-30

**Authors:** Justas Ciganas, Paulius Griskevicius, Arvydas Palevicius, Sigita Urbaite, Giedrius Janusas

**Affiliations:** Department of Mechanical Engineering, Kaunas University of Technology, Studentu 56, 51424 Kaunas, Lithuania

**Keywords:** microstructure, finite element simulation, thermal imprint, polymer

## Abstract

Polymeric microstructures and microchannels are widely used in biomedical devices, optics, microfluidics and fiber optics. The quality, the shape, the spacing and the curvature of microstructure gratings are influenced by different mechanisms and fabrication techniques used. This paper demonstrates a cost-effective way for patterning high-aspect-ratio thermoplastic microstructures using thermal imprint technology and finite element modeling. Polymeric materials polypropylene (PP), polyethylene terephthalate glycol (PETG), polyvinyl chloride (PVC) and styrene-acrylonitrile (SAN) were chosen for the experimental investigations. A finite element model was constructed to define the most suitable parameters (time, heating temperature, pressure, etc.) for the formation of microstructures using the thermal imprint procedure. To confirm the relevance of the finite element model, different types of PP, PETG, PVC and SAN microstructures were fabricated using theoretically defined parameters. Experimental investigations of imprinted microstructures’ morphological and optical properties were performed using scanning electron microscopy, atomic force microscopy and a diffractometer. Obtained results confirmed the relevance of the created finite element model which was applied in the formation of high-aspect-ratio microstructures. Application of this model in thermal imprint would not only reduce the fabrication time, but also would highly increase the surface quality and optical properties of the formed structures.

## 1. Introduction

In recent years, novel micro- and nanomanufacturing technologies have been intensively developed for fabrication of microstructures [1]. Different applications require different grating parameters and materials to be used (shape, size and structure) in microfluidics. The developed microstructures are used in different applications, such as biosensors, wavelength division multiplexing devices, optical devices, nano- and microfluidics, electronics, sample separation or single-molecule analysis [2,3,4,5], to transport particles or materials, separate, dispense or mix liquids [6].

Because of the precision required for fabrication of microchannels, the lack of suitable and effective conventional technologies is hindering the development of microstructure-based devices. Processes such as photolithography, laser processing or etching are conventional, but mostly time-consuming grating manufacturing technologies. A thermal imprint technology is one of the possible choices for forming microstructures in thermoplastics at a relatively high speed and low cost. Basic advantages of this technique include low material flow and low flaw rates, which allows to avoid internal stress resulting in more delicate gratings [7]. The mostly used thermoplastics for fabrication of microchannels are: polypropylene (PP), polyethylene terephthalate glycol (PETG), polyvinyl chloride (PVC) and styrene-acrylonitrile (SAN). PP thermoplastic is distinguished by being biocompatible and is mostly used in MEMS devices such as thermal microactuators [8]. PETG plastic has a higher glass transition temperature (T_g_), meaning that a formed microstructure can encounter higher temperatures. In some cases, PETG thermoplastic is used as a template for embossing in plastics with low glass transition temperature [9]. PVC microstructural film can be used as an anti-reflection layer on solar cells with a high efficiency [10]. The glass transition temperature of SAN thermoplastic is higher than 100 °C, which makes the material resistant to boiling water [11]. Thus, selected plastics are widely used in thermal printing and have different applicability in MEMS devices.

However, to create high-aspect-ratio microstructures and microchannels using the thermal imprint technique, it is necessary to study the behavior of the materials in the plastic deformation stage. High-aspect-ratio microchannels need to be designed in order to avoid uneven flow of the liquid, to achieve a larger active surface area, to create massive microdevices with parallelization functionality or to achieve higher system throughput. Thus, finite element modeling was used in order to optimize and better understand structural changes in thermoplastics and analysis of the thermomechanical changes throughout the process. It allowed to define the optimal thermal imprint parameters of the plastics (PP, PETG, PVC and SAN) used in the formation of gratings on the surface. This paper covers the numerical simulation and experimental investigations used to find the basic parameters for formation of microstructures on different types of thermoplastics, PP, PETG, PVC and SAN. Evaluation of thermoplastic properties and tensile experiment showed the behavior of PP, PETG, PVC and SAN under stress. Obtained results were used in the interpolation of temperature, stress and strain, i.e., the properties of the plastics were determined by multilinear isotropic hardening using finite element modeling. Further, determined parameters were used in thermal imprint procedures to form qualitative microstructures of four different thermoplastics. Surface properties were evaluated using scanning electron microscopy (SEM) and atomic force microscopy (AFM), and optical properties were analyzed using a He-Ne laser diffractometer.

## 2. Materials and Methods

***Materials.*** Four thermoplastics, polypropylene (PP), polyethylene terephthalate glycol (PETG), polyvinyl chloride (PVC) and styrene-acrylonitrile (SAN), were used for the investigations. Theoretical material characteristics of PP, PETG, PVC and SAN are given in Table 1, including Young’s Modulus, Poisson’s Ratio, Bulk Modulus, etc.

For analysis of geometrical behavior of formed microstructures using the thermal imprint technique, it is necessary to define additional properties of investigated thermoplastics. Deformations of the elastic part can be described by Young’s modulus, but additional data are required at the onset of plastic deformation, i.e., multilinear isotropic hardening.

***Tensile testing*.** Behavior of thermoplastics in the plastic deformation stage can be described in several ways. One of the most popular, the Mooney–Rivlin model, was used in these investigations. So, first of all, experiments on tensile tests of four thermoplastics were performed at room temperature, i.e., analysis of materials’ behavior as the temperature reaches the glass transition temperature in order to observe the direct multilinear isotropic hardening.

**Figure 1:**
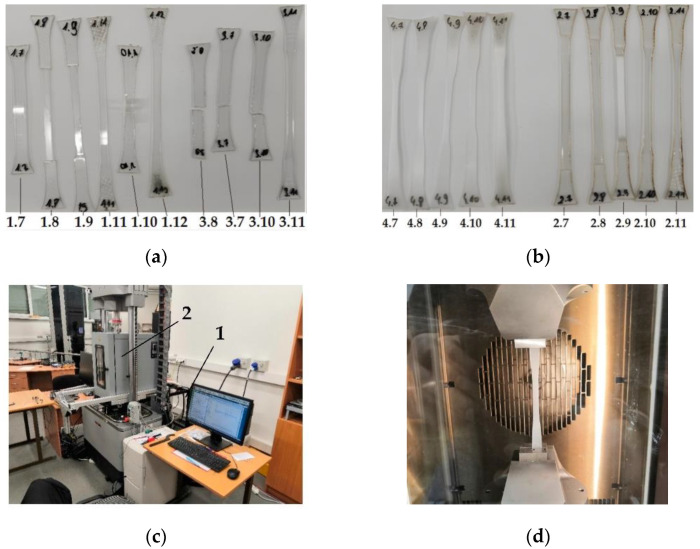
Tensile experiment. Specimens after the experiment: (**a**) 1.7–1.12—PVC, 3.7–3.11—SAN; (**b**) 4.7–4.11—PP, 2.7–2.11—PETG; (**c**) Testing machine Instron E10000: 1—control computer, 2—heating chamber and testing machine; (**d**) The test sample in the grippers placed in the heating chamber.

The tensile experiment was performed with an Instron E10000 test apparatus (Figure 1c). The test machine consists of standard tensile components and an additional thermal chamber (Figure 1c), which was used to create the temperature medium. Standard ISO 527-2 dog bone specimens were made from PP, PETG, PVC and SAN laser-cut sheet plastic. Each thermoplastic was prepared in five blanks that were stretched at different temperatures (Figure 1a,b). All the samples were stretched out (Figure 1d), except the specimens made from SAN (numbered as 3.7–3.10). Since SAN is brittle, after reaching the glass transition temperature, the thermoplastic changed its properties.

**Figure 2:**
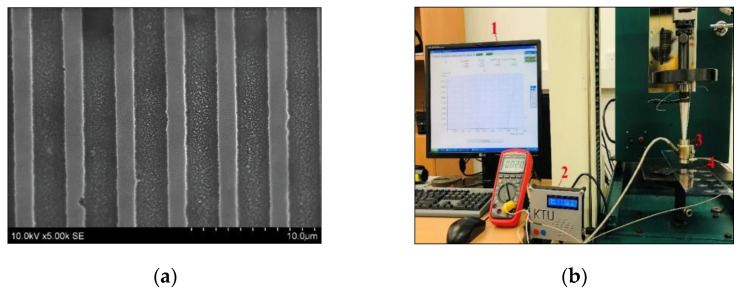
(**a**) SEM image of the master matrices. (**b**) Tensile testing experimental equipment: 1—a computer that controls the test machine, 2—temperature controller, 3—cathode element, 4—sample.

***Thermal imprint*.** A master grating for formation of microstructures on analyzed thermoplastics was fabricated by lithography and reactive ion etching technologies, using crystalline silicon material deposited on the nickel base. Thus, a fabricated microstructure consisted of a series of two-dimensional grooves, with defined parameters of 2 µm width, 4 µm periodicity and 1 µm depth (Figure 2a).

A ‘Tinius Olsen’ test machine, together with a punch (in this case, the basis for the microstructure), the heating element and the controller were used for the fabrication of different thermoplastics’ microstructures (Figure 2b). Before starting the embossing process, a mold with a thermoplastic plate was first heated to a defined temperature and allowed to stabilize. The force, temperature and time acting on the structure were varied in response to the resulting structure view. Accuracy of the machine was within ±0.5% of the indicated load from 0.2% to 100% of capacity.

***Atomic Force Microscope*.** Surface morphology was investigated using Atomic Force Microscope NT-206 in the static/dynamic mode at 10 μm/s.

**Figure 3:**
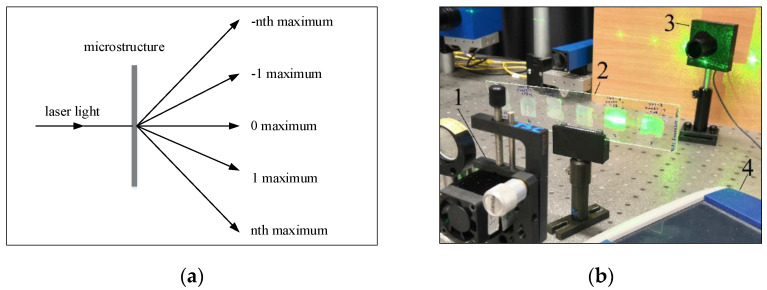
Diffraction efficiency measurements: (**a**) structural image of the diffraction peaks; (**b**) measurement equipment: 1—laser, 2—grid, 3—photodiode, 4—a visual display of results.

**Figure 4:**
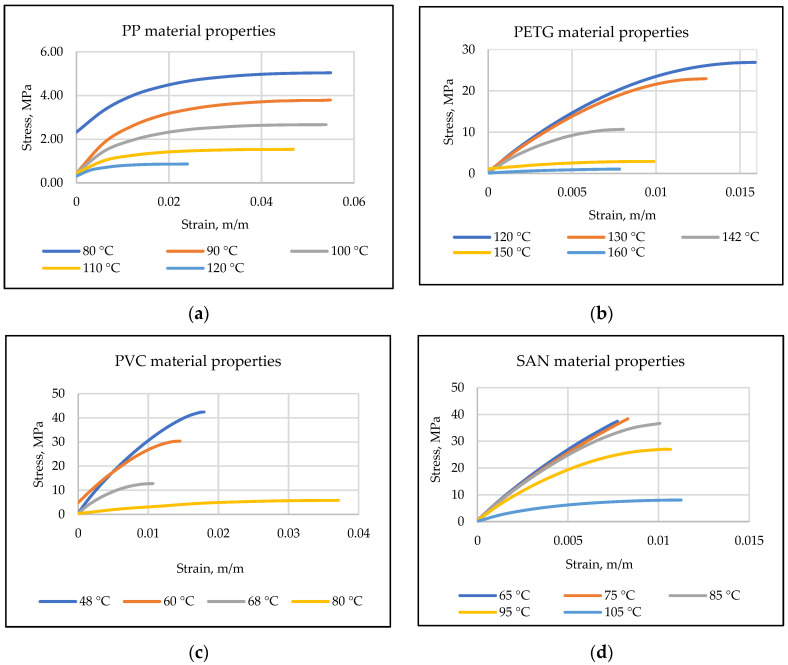
Stress and strain curves of (**a**) PP, (**b**) PETG, (**c**) PVC and (**d**) SAN thermoplastics.

***Diffractometer.*** Diffraction efficiency measurements at different peaks (Figure 3a) were performed using a He-Ne laser diffractometer system (Figure 3b) to measure the efficiency at all peaks of the microstructure. Thus, samples were mounted on a rack (Figure 3b and Figure 2) and illuminated with a green laser light of wavelength 532 nm. An Extreme Low Power Laser Detector 11XLP12-3S-H2 (Figure 3b, element 3) was used for the low power µW regime with very low thermal drift and a repeatability of ±0.5%, calibration uncertainty ±2.5% and sensitivity of 200 mV/W. Thus, transmitted light peaks were collected by a photodiode and measured with a Maestro energy monitor (Figure 3b and Figure 4).

Basically, the efficiency of the microstructure depends on the geometry and the quality of the formed gratings. One of the main parameters evaluating the optical properties of a structure is relative diffraction efficiency (RDE), which can be calculated by the equation:

(1) $${\mathrm{RDE}}_{i,j}=\frac{P_{i,j}}{\sum_{i}P_{i,j}}$$

where ${\mathrm{RDE}}_{i,j}$ is the relative diffraction efficiency and $P_{i,j}$ is the light power intensity of the maximum.

## 3. Results

### 3.1. Tensile Testing of Termoplastics

The specimens were stretched until they were broken or the maximum limits of the test machine were reached. One of the grippers was moving at a constant speed of 20 mm/min. The thermal integrity of the samples was ensured by heating, i.e., the samples were allowed to heat up and then to stabilize as the temperature in the chamber changed. Registered experimental stress–strain curves for different thermoplastics are given in Figure 4.

Using obtained stress–strain curves and the tangent lines, passing through the slopes, Young’s modulus for each thermoplastic was calculated defining the behavior of the material under the stress and results are given in Table 2. Thus, increasing the temperature lead to decreasing Young’s modulus for different plastics.

Experimental results, given in Table 2, showed that, for PP thermoplastic, the temperature range was chosen from 80 to 120 °C and Young’s modulus decreased, respectively, with increasing temperature. During the experiment with PETG thermoplastic, because of the temperature change, PETG suddenly changed its color and became matte (matting appeared at 142 °C when the thermoplastic began to stretch). Temperature limits for PVC thermoplastic were from 48 °C to 90 °C, however, at 90 °C, it did not have enough acceleration to exceed the stress limit, i.e., PVC thermoplastic relaxed more quickly due to temperature than stresses formed. Finally, for SAN, the temperature range was chosen from 65 °C to 105 °C, thus, Young’s modulus decreased with increasing temperature until the sample broke. Working temperature ranges were chosen according to the thermoplastics’ theoretical thermal properties.

Thus, experimental results show that temperature changes have a significant influence on Young’s modulus, i.e., decreasing with increasing temperature. These results can be interpolated to find intermediate Young’s modulus values.

### 3.2. Numerical Simulation of Thermal Imprint

Finite element modeling was used to investigate the geometrical behavior during the thermal imprint of PP, PETG, PVC and SAN. The designed model analyzed the hot stamping technology at the micro-level using the parameters defined during the tensile testing (given in Table 2).

**Figure 5:**
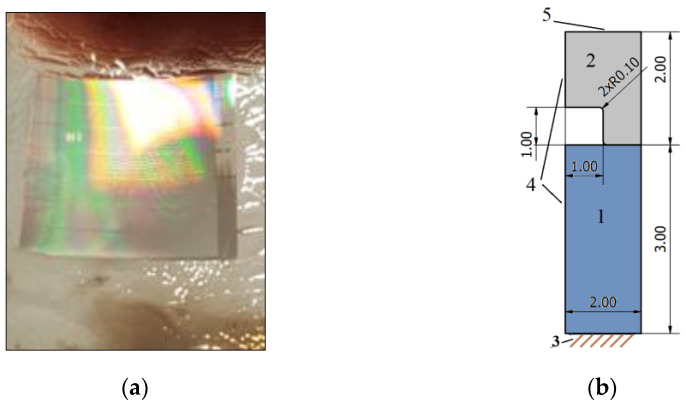
(**a**) Template of nickel master grating plate; (**b**) drawing of one master-grating element with boundary conditions: 1—substrate, 2—stamp, 3—fixed support, 4—symmetry region and 5—an external force.

In the numerical simulation, parameters of the fabricated nickel master grating with two-dimensional grooves of 2 µm width, 4 µm periodicity and 1 µm depth (Figure 5) were used.

**Figure 6:**
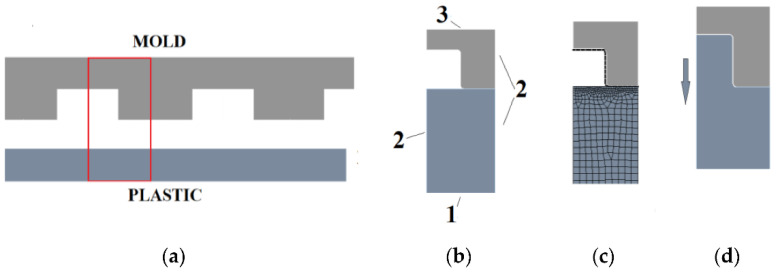
Simplified microstructure. (**a**) Cross-sectional shape of the mold and thermoplastic substrate; (**b**) analyzed model: 1—fixed support, 2—frictionless support and 3—remote displacement; (**c**) meshing of the model; (**d**) deformed analyzed model.

Further, ANSYS software was used for the finite element model and the geometry of the microstructure with a mold (or master grating) (Figure 6a) was simplified to half of single element of master grating, and defined under boundary conditions (Figure 6b). Application of the Mooney–Rivlin model, when the glass transition temperature (T_g_) was reached or exceeded, was used to analyze and simulate the thermoplastic properties of PP, PETG, PVC and SAN obtained during the experimental research. Defined properties were used in the interpolation of the temperature, stress and strain. Thus, in the finite element model, the properties of the materials were determined by multilinear isotropic hardening.

**Figure 7:**
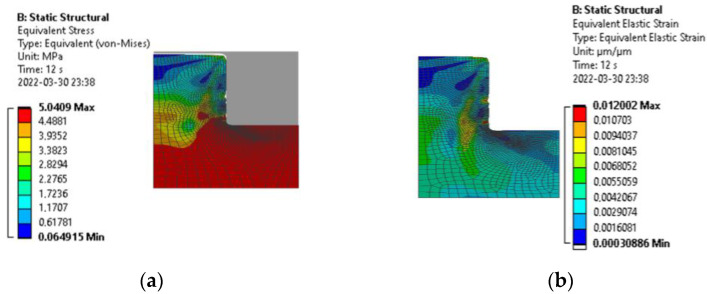
Stress and strain distribution: (**a**) Equivalent stress legend and stresses at maximum strain; (**b**) residual stress image with the equivalent elastic strain legend.

The created mathematical model consists of a flexible thermoplastic (in stiffness) and a non-deformable mold. It was described as two-dimensional and the parameters were defined as 1 mm long. Frictional contact with a 0.2 friction coefficient was used between thermoplastic and metal. Thus, the coefficient of friction, due to plastic deformations and constraints, is not significant in these investigations [15]. The model was divided into 0.2 µm elements, but in the contact areas the scale was reduced to 0.05 µm (Figure 6c). In the simulation, the plastic base was rigidly fixed. Frictionless support was used to assess the integrity of the model. The mold moved toward the plastic in automatic steps up to 2.25 µm and then retracted (Figure 6d). A nonlinear adaptive region was used to modify the grid during formation in order to obtain more accurate results. Output data for total deformation, equivalent elastic strain, equivalent stress and reaction were selected to obtain the plot of stress and strain (Figure 7).

The grid-independent verification study showed that the influence of the grid is not significant in these calculations. The results of strain, stress and reaction did not significantly change after grid compaction. The mesh can be of coarse quality to optimize the calculations (Table 3).

After all simulations, the maximum stresses, strains and reaction forces were found (Table 4). The reaction force was calculated for an area of 2000 µm^2^. To maintain the same pressure, the total force required for a full mold embossing must be increased, respectively.

Thus, the simulation result (Table 4) proved the relevance of the finite element model because the maximum stresses reached the maximum described limits in all cases. Thus, the reaction force decreased steadily with decreasing stresses. Further, a created finite element model will be applied in the formation of high-aspect-ratio microstructures, i.e., applying theoretically determined forces during the thermal imprint process would not only reduce the fabrication time but also would highly increase the quality of the formed structures. Knowing the properties of the material helps to find out what reaction force is needed to perform thermal imprint. So, the simplification of the model optimizes the calculation time and allows to obtain the highest quality and repeatability of the deformable plastics during the thermal imprint procedure.

### 3.3. Thermal Imprint Process Based on Finite Element Modeling Data

**Figure 8:**
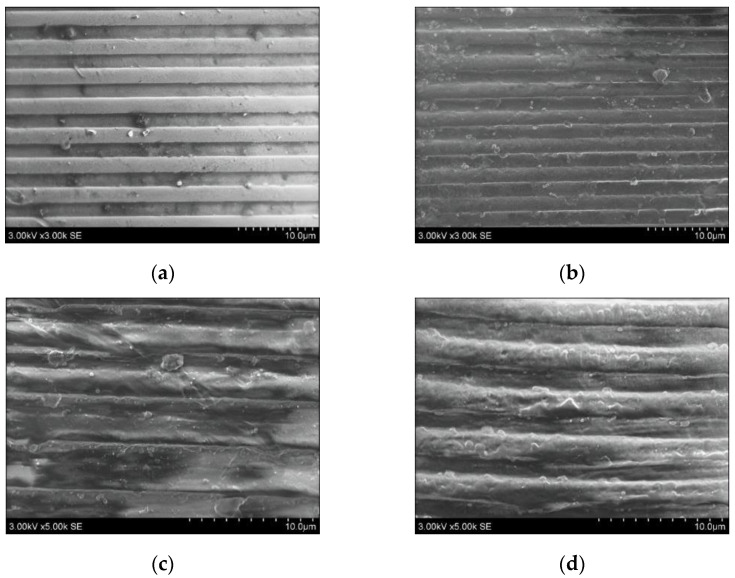
Formation of microstructures: (**a**) microstructure formed in PETG; (**b**) microstructure formed in PP; (**c**) microstructure formed in PVC; (**d**) microstructure formed in SAN.

To prove the relevance of the created finite element model, fabrication of PP, PETG, PVC and SAN microstructures, using defined theoretical parameters, was performed. A numerical simulation allowed to determine the exact value required for the formation of a microstructure during thermal imprint technology. In the experiment, a ‘Tinius Olsen’ test machine (Figure 2b) with a nickel (Ni) master grating (Figure 2a) was used. By varying the force, temperature and time during the process, microstructures in thermoplastics were imprinted (Figure 8a–d).

Thus, microstructures were embossed in all four thermoplastics—PP, PETG, PVC and SAN—and their 3D views were made using a scanning electron microscope (SEM) (Figure 8). Thus, PETG and PP showed higher-quality reproduction and smoother surfaces than PVC and SAN thermoplastic microstructures.

#### 3.3.1. Surface Morphology of Imprinted Microstructures

**Figure 9:**
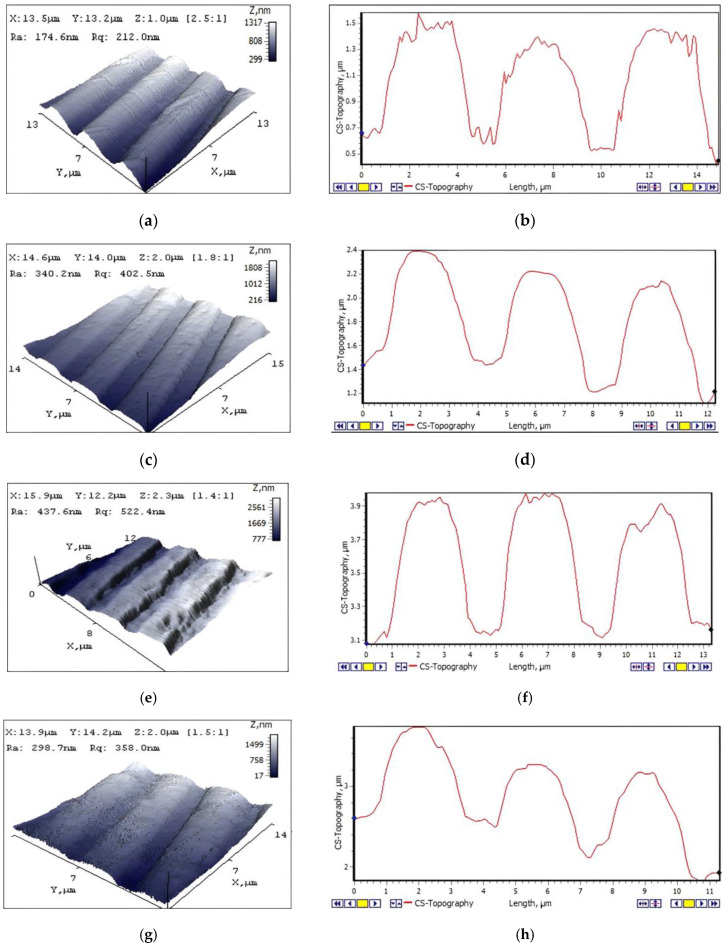

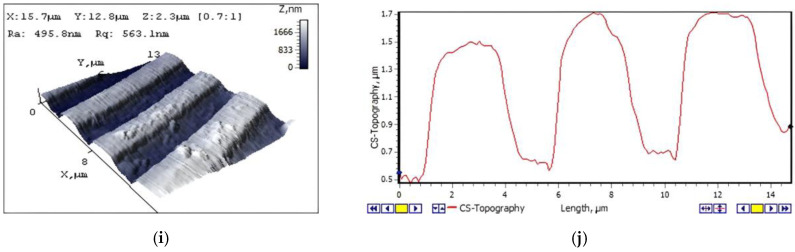
AFM topography images and surface profiles of embossed microstructures in thermoplastics with topography: (**a**,**b**) PETG; (**c**,**d**) PP; (**e**,**f**) PVC; (**g**,**h**) SAN; and (**i**,**j**) a nickel master grating.

Surface view and geometry of the microstructures’ topographic profiles were examined using an atomic force microscope. Best results were obtained of the microstructure formed in PETG thermoplastic (Figure 9a,b): a rather smooth surface with the average surface roughness Ra = 174.6 nm and an average grating depth of 400 ± 20 nm. The microstructure from PP thermoplastic had a smooth surface relief of average surface roughness Ra = 340.2 nm with a geometry similar to master grating with an average grating depth of 330 ± 30 nm (Figure 9c,d). PVC and SAN microstructures had many defects on the surface with an average roughness of Ra = 437.6 nm and Ra = 298.7 nm, respectively (Figure 9e,g). From profile views, it is seen that the geometry of the gratings is uneven in depth, width and the form itself (Figure 9f,h), compared to parameters of nickel master grating (Figure 9i,j).

Thus, results of SEM and AFM measurements imply that the most qualitative microstructures were obtained from PP and PETG thermoplastics. This might influence not only on the thermal imprint procedure, but also on the thermal properties of the plastics themselves. Thus, PVC and SAN are more brittle materials compared to PP and PETG, and the results of the imprinted microstructures were less accurate.

#### 3.3.2. Optical Properties of Imprinted Microstructures

The quality of microstructures is defined not only by geometrical parameters of the formed gratings, but also by optical properties. In this paper, diffraction efficiency measurements in different peaks were performed using a He-Ne laser diffractometer. Due to the non-optical nature, the PP material was not examined. So, only diffraction efficiencies of PETG, PVC and SAN microstructures were measured and evaluated.

Measurements of diffraction efficiency were performed for 12 samples of each thermoplastic, fabricated at different embossing conditions (load, embossing time and temperature) (Table 5). Thus, the best relative diffraction efficiency RDE = 34.62% was observed in the PETG microstructure, fabricated at the following embossing conditions: load of 2000 N for 10 s at 125 °C (Table 5). For the SAN microstructure, the best values of RDE = 29.04% were obtained when embossing parameters were 5000 N for 10 s at 130 °C. The PVC microstructure showed best results of RDE = 22.44% when a load of 5000 N was applied for 10 s at 80 °C during a thermal imprint procedure. Applying theoretical calculations and experimental maximum diffraction efficiency of plastics, the grating depth of the plastics were obtained: SAN—0.62 µm, PETG—0.57 µm and PVC—0.72 µm. The relative error between the measured spectral diffraction efficiencies using the diffractometer is 1.24% in the case of SAN, 1.13% in the case of PETG and for PVC the relative error is 1.57%. The load and time are constant values during the hot imprint process.

**Figure 10:**
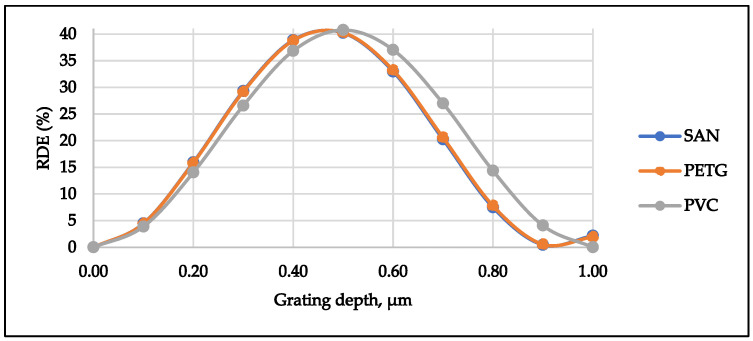
Dependence of the theoretical diffraction efficiency of plastics on the grating depth.

To prove the relevance of the experimental results, theoretical diffraction efficiencies of microstructures were calculated. The following refractive indexes of plastics were used for the calculations: SAN—1.572 [16], PETG—1.57 [17] and PVC—1.531 [18]. The direct influence of the grating depth on diffraction efficiency was determined. Thus, results implied that the grating depth can be determined from the diffraction efficiencies using the theoretical calculations (Figure 10).

It may be concluded that the diffraction efficiency of all microstructures (PETG, PVC and SAN) was higher when the temperature exceeded the plastic glass transition temperature, i.e., exceeding the glass transition temperature impairs the properties of thermoplastics.

## 4. Discussion

Microfluidics requires high-aspect-ratio microchannels to be designed in order to avoid uneven flow of the liquid, to achieve a larger active surface area, to create a massive MEMS device with parallelization functionality or to achieve higher system throughput [19,20]. Using the thermal imprint procedure, there are some important factors which should be evaluated in advance, and final element modeling is a great way to do it. The advantages of the finite element model give the opportunity to change the geometry, i.e., in this case, it is much easier to find the parameters required for forming qualitatively replicated structures by the hot imprint procedure. Thus, the results confirmed that is better to form the microstructure when the temperature is near the glass transition temperature and when the plastic can flow slowly into the mold. Time, here, also plays an important role leading to better surface morphology and optical properties.

## 5. Conclusions

The behavior of thermoplastics can be simulated using multilinear isotropic hardening and applying the created finite element model. Using the results of the tensile experiment, successfully interpolated stress and strain distributions at different temperatures of PP, PETG, PVC and SAN thermoplastics were obtained.

Using the numerical simulations, optimal working parameters, stress and strain distribution were defined and applied in the thermal imprint process for formation of microstructures. AFM results showed that the PETG thermoplastic microstructure had the best surface properties compared to other PP, PVC and SAN microstructures. Its optical properties were proved with diffraction efficiency measurements, which showed the best quality of the PETG microstructure under the following conditions: grating formation under load of 2000 N for 10 s at 125 °C. Thus, the best diffraction efficiency measurement results for the PVC microstructure were observed when it was formed at 5000 N for 10 s at 80 °C. For the SAN microstructure, the best results were obtained when it was formed under a load of 5000 N for 10 s at 130 °C.

The created finite element model is a useful tool for fabrication of high-aspect-ratio microstructures with great surface morphological and optical properties. The model may easily help to control the thermal imprint process to ensure the quality of microstructures when slow formation occurs.

## Figures and Tables

**Table 1:** Properties of PP, PETG, PVC and SAN plastics [12,13,14].

	PP	PETG	PVC	SAN
**Young’s Modulus (MPa)**	1325.0	2950.0	3275.0	3650.0
**Poisson’s Ratio**	0.43	0.4	0.4	0.4
**Bulk Modulus (MPa)**	3888.9	4868.0	5458.3	4055.6
**Shear Modulus (MPa)**	1296.3	1054.3	1169.6	1351.9
**Isotropic Secant Coefficient of Thermal Expansion (1/°C)**	6.8 × 10^−5^	4.3 × 10^−5^	3 × 10^−5^	6.8 × 10^−5^
**Tensile Ultimate Strength (MPa)**	32.94	67.4	52.0	85.0
**Tensile Yield Strength (MPa)**	26.1	58.7	54.8	83.4

**Table 2:** Young’s modulus of PP, PETG, PVC and SAN thermoplastics at different temperatures.

**PP**	**Temperature, °C**	**80**	**90**	**100**	**110**	**120**
	Young’s modulus, MPa	420.0	320.0	240.0	152.0	113.3
**PETG**	**Temperature, °C**	**120**	**130**	**142**	**150**	**160**
	Young’s modulus, MPa	3125.0	3000.0	2666.7	333.3	250.0
**PVC**	**Temperature, °C**	**48**	**60**	**68**	**80**	**90**
	Young’s modulus, MPa	3750.0	2916.7	2333.3	187.5	-
**SAN**	**Temperature, °C**	**65**	**75**	**85**	**95**	**105**
	Young’s modulus, MPa	6000.0	5692.3	5142.9	4222.2	1846.1

**Table 3:** The grid-independent verification.

	Elements	Strain, µm/µm	Stress, MPa	Reaction, µN
**Coarse**	1533	1.2107 × 10^−2^	5.0409	16,655
**Medium**	2873	1.2383 × 10^−2^	5.2205	16,701
**Fine**	5129	1.2089 × 10^−2^	5.2511	16,647

**Table 4:** Simulation results of thermoplastics’ behavior during thermal imprint.

**PP**	**Temperature, °C**	**80**	**90**	**100**	**110**	**120**
	Stress, MPa	5.0409	3.793	2.668	1.5368	0.8648
	Strain, µm/µm	0.012	0.011	0.011	0.010	0.0076
	Reaction force, µN MAX	16,655	12,458	8736	5024.9	2804.8
**PETG**	**Temperature, °C**	**120**	**130**	**142**	**150**	**160**
	Stress, MPa	26.923	22.966	10.703	2.927	1.0588
	Strain, µm/µm	0.0086	0.00765	0.00401	0.00878	0.00423
	Reaction force, µN MAX	87,723	74,711	35,215	9585	3454.8
**PVC**	**Temperature, °C**	**48**	**60**	**68**	**80**	**90**
	Stress, MPa	42.387	30.351	12.75	5.793	-
	Strain, µm/µm	0.0113	0.0104	0.00546	0.0308	-
	Reaction force, µN MAX	138,500	99,140	41,886	18,974	-
**SAN**	**Temperature, °C**	**65**	**75**	**85**	**95**	**105**
	Stress, MPa	37.457	38.379	36.66	26.987	8.0495
	Strain, µm/µm	0.0062	0.0067	0.0071	0.00639	0.00436
	Reaction force, µN MAX	125,080	126,100	119,700	88,337	26,436

**Table 5:** Diffraction efficiency measurement results.

SAN				PETG				PVC			
Load (N)	Time (Sec)	Temp. (°C)	RDE (%)	Load (N)	Time (Sec)	Temp. (°C)	RDE (%)	Load (N)	Time (Sec)	Temp. (°C)	RDE (%)
5000	10	100	27.07	4000	10	100	24.31	5000	10	100	5.39
5000	10	120	26.49	5000	5	100	31.43	5000	5	100	12.63
5000	15	120	24.94	5000	10	100	30.50	4000	10	100	7.28
5000	10	140	22.52	4000	5	100	23.49	4000	5	100	11.59
4000	10	140	23.28	**2000**	**10**	**125**	**34.62**	3000	10	100	5.43
4000	5	140	17.85	2000	10	100	28.81	3000	5	100	11.66
3000	10	140	28.12	2000	5	100	32.31	4000	10	125	10.44
3000	5	140	26.85	3000	10	100	30.67	4000	5	125	7.38
3000	10	130	28.23	2000	10	90	24.85	5000	5	90	6.03
5000	2	130	27.76	2000	5	90	29.86	**5000**	**10**	**80**	**22.44**
**5000**	**10**	**130**	**29.04**	2000	10	80	23.54	2000	10	125	6.74
4000	10	130	25.25					2000	5	125	6.65
